# COVID-19, people with disabilities, and the Italian government recovery: investigating the impact and promoting psychological resources to prevent future emergencies

**DOI:** 10.3389/fpsyg.2023.1260853

**Published:** 2023-10-25

**Authors:** Elisabetta Camussi, Daria Meneghetti, Maria Luisa Sbarra, Riccardo Rella, Francesca Barillà, Cinzia Sassi, Lorenzo Montali, Chiara Annovazzi

**Affiliations:** ^1^Department of Psychology, University of Milan-Bicocca, Milan, Italy; ^2^Department of Human and Social Sciences, University of Valle D’Aosta, Aosta, Italy

**Keywords:** people with disabilities, COVID-19, Life Design, wellbeing, resilience, future orientation, career adaptability, life satisfaction

## Abstract

**Introduction:**

Given its profound and transversal impact, the COVID-19 pandemic in 2020 marked a deep point of division in how people make sense of the world and their lives. The consequences of this event were remarkable, especially for populations already facing vulnerability, exclusion, and discrimination. In Italy, over 3 million people (5.2% of the entire population) have a disability due to health issues or severe limitations that prevent them from performing daily activities. Although the COVID-19 health emergency aggravated and amplified these problems, research and studies investigating the incidence of psychological distress and the role of psychological resources for people with disabilities in the aftermath of the pandemic are still to be implemented. For these reasons, the Department of Psychology conducted a study on behalf of the Italian Government to assess the impacts of the COVID-19 pandemic on the social, psychological, and economic wellbeing of Italians with disabilities.

**Methods:**

The aim was to assess the consequences of the pandemic on this population, especially the impacts related to the lockdowns and preventive measures, and to evaluate the protective role that could be played by psychological resources such as resilience, future orientation, and career adaptability in a Life Design perspective. With the collaboration of local, regional, and national associations for people with disability, an anonymous, online self-report questionnaire was distributed to 403 persons with disabilities in Italy.

**Results:**

Results showed a strong relationship between the levels of psychological resources and life satisfaction during the COVID-19 pandemic.

**Discussion:**

In line with studies in international literature regarding the effects of the COVID-19 pandemic on people with disabilities, this research highlights the extension of this period’s impacts on this population’s psychological wellbeing. Moreover, this study amplifies the urgent call for action and research in promoting Life Design psychological resources, given their positive and protective role in preserving and increasing people’s wellbeing.

## Introduction

In early December 2019, a cluster of pneumonia cases was detected in Wuhan, China, followed by a rapid spread across the country and the world. Epidemiological studies identified three conditions related to the virus spread: source of infection, route of transmission, and susceptibility (Wang L. et al., 2020). SARS-CoV-2 was a surprisingly highly transmissible and rapidly spreading virus, and these characteristics had an immediate effect on the world population by transversely affecting people of different ages, genders, and health conditions. Although dangerous viruses have been threatening humankind for millennia, pandemics have been very rarely studied, and this aspect may have been one of the reasons for the low level of professional preparedness to counter COVID-19 (Roehrle, 2020). Unlike other 21st-century virus epidemics, such as SARS and MERS, which spread primarily in hospital settings (Bai et al., 2004; Cauchemez et al., 2016), COVID-19 quickly crossed the boundaries of health centers putting the entire population at risk. The number of cases of people with COVID-19 grew so dramatically that on March 11, 2020, the World Health Organization, after assessing the severity levels and global spread of SARS-CoV-2 infection, declared a pandemic (ISS, 2020). The consequences of this were global, severely affecting the world in terms of quality of life, as well as environmental and sustainable economic development (El Keshky et al., 2020). At the societal level, the COVID-19 pandemic was perceived as highly threatening and affected the collective perception and imagination through media coverage and reports of virus progress and mortality rates (Roehrle, 2020). Cognitive processing of such stressful events has a significant impact on individuals’ emotional responses and overall wellbeing (Diao et al., 2023), and the necessary restrictive measures created an unprecedented scenario dominated by fear and uncertainty. The restrictions implemented to counter and contain the COVID-19 pandemic triggered a spiraling and negative effects on food and consumer supply chains due to restrictions on the movement of cross-border transportation in most countries with a negative impact on all global economies. As pointed out in a study by Allam et al. (2022), the interconnected nature of supply chains will make them even more vulnerable, not only because of the long-term consequences of COVID-19, but also due to the cascading impacts of conflicts resulting from the ongoing war between Ukraine and Russia, the resulting shortages and scarcities of various products (particularly consumer goods, food, and oil and energy products) and related European security and energy issues (Bond et al., 2022). At an economic level, there has been a decline in global GDP, capital flows, reduced investment opportunities, and decreased trade. The pandemic also set in motion the third and most severe economic and social crisis since the beginning of the new millennium, following September 11, 2001, and the recession of 2007–2011 (Salustri, 2020). The crisis triggered by COVID-19 differs from the situation of 2007–2011 in its exogenous and totally unforeseen origin compared to the trends in the economic-financial system, and in its effects on national and global economies. In Italy, the impact of the health crisis hit the economy particularly acutely, with a fall in GDP of 8.9 percent in 2020, determined essentially by the collapse of domestic demand and especially consumption. The crisis also affected the labor market: initially, the decline in employment mainly concerned term employees and the self-employed, then also permanent workers. As of April 2021, compared to before the emergency, employment had declined by more than 800,000 (ISTAT, 2021). In Italy, demographic dynamics, the postponement of life cycle stages, the spread of precariousness and the fragmentation of work paths, as well as reduced levels of social mobility have contributed to undermining the chances of realization of opportunities for a large proportion of young people and to discouraging their participation at the political, social, and cultural levels (ISTAT, 2023). This situation affects both the unemployment rate and the quality of work, and the progress achieved over the past decade in terms of poverty reduction has been wiped out by the COVID-19 crisis. According to the new International Labor Organization Report (ILO, 2023), the current global economic slowdown is likely to force more workers to accept lower-quality, lower-paid work with little stability and social protection, thus accentuating the inequalities that have multiplied during the COVID-19 crisis. Therefore, the pandemic and post-emergency period have an impact at various levels and on different aspects of people’s lives with consequent implications for wellbeing and mental health, as will be more fully explained in the following sections.

### The impact of COVID-19 on psychological and social wellbeing

The COVID-19 pandemic period has accentuated the already relevant global dimensions of changeability, non-linearity, and instability, leading individuals to lose stable reference points, increasing the sense of uncertainty, anomie, and concern (Dryhurst et al., 2020). Separation from the non-cohabiting family, loneliness, school closures, financial insecurity, job uncertainty, and the stigmatization of people who tested positive for COVID-19 led to the development of critical issues with repercussions on the level of wellbeing and quality of life for both individuals and communities (Sood, 2020). With 16 million infections and over 160 thousand deaths associated with SARS-CoV-2 infection between March 2020 and April 2022, Italy was, together with Spain, among the EU countries most affected by the pandemic. The worst period coincided with the first lockdown phase (March–May 2020), with marked improvements from 2021, in conjunction with the beginning of the first vaccination campaign (ISTAT, 2022). From a clinical point of view, the pandemic favored the development of anxiety disorders in most of the population, regardless of virus contraction and/or disease development, progression, and medical consequences. Furthermore, people had to deal with isolation from their loved ones, which turned out to be highly dysfunctional in interpersonal relationships and mental health (Farooq et al., 2020). Women, young people, and immigrants were confirmed as the most fragile subjects, together with people with disabilities and their families. Precariousness regarding the future, intense health concerns, difficulty finding gratification, and, in many cases, depressive undertones led to a severe diminishing in the quality of life of Italian people (Epifanio et al., 2021). Indeed, data show an evident worsening in the physical, psychological, relational, and environmental wellbeing of the population. The variables most associated with this deterioration in life conditions are female gender, young age, unemployment, a low socio-cultural level, the area of residence, and the diagnosis of a medical-psychiatric condition. Finally, the psychological attitude with which the COVID-19 emergency was faced also plays a major role. People who were most dissatisfied with the quality of their lives were, in fact, those who showed a higher sense of helplessness regarding the possibility of containing the spread of the infection by complying to the rules of hygiene and social distancing (Epifanio et al., 2021). Even the most recent data about the new “post-pandemic” period are mostly worrying, pointing out negative consequences especially at a psychological level (ISS, 2023). In line with this, Sutin et al. (2022) emphasize the burden the pandemic had on people, especially the younger ones: “young adults experienced a kind of interrupted maturity, coupled with an increase in neuroticism and a parallel decrease in agreeableness and conscientiousness.” In addition to this, as more widely depicted in the next paragraph, according to an extensive survey conducted in Europe by Ahrendt via Eurofound (2022), disability is a key factor in indicating an aggravated suffering from many points of view during and after the COVID-19 pandemic.

### The impact of COVID-19 on people with disabilities

From the first lockdown, the COVID-19 emergency inevitably led to significant repercussions on people with vulnerability, disabilities, and conditions of fragility (World Health Organization, 2020). These negative consequences especially concerned income, health, education, and an increase in inequality (ISTAT, 2022). According to the United Nations High Commissioner for Human Rights (UNHCR), “while the pandemic threatens all members of society, people with disabilities suffer a disproportionate impact due to attitudinal, environmental and institutional barriers which are also reproduced in the response provided to the COVID-19” (United Nations High Commissioner for Human Rights, 2020). During the COVID-19 pandemic, people with intellectual and relational disabilities were particularly affected, given the heightened difficulties in making sense of the pandemic and of the precautions to be taken, such as home confinement or the use of face masks. Many people with disabilities who rely daily on others (through formal or informal support) found themselves lacking the support they needed due to movement restrictions and social distancing measures. This hindered access to food, essential goods, and medicines, and limited the performance of basic daily activities such as bathing, cooking, or eating (Croft and Fraser, 2022). The interruption of care and support also jeopardized the work situation of caregivers. They were often forced to give up or limit their jobs to devote themselves to care work, resulting in financial difficulties. From a physical health point of view, the arrival of COVID-19 increased the difficulties in performing and planning medical assessment procedures. Indeed, two-thirds of health appointments were postponed. At the same time, almost half (43%) of annual health reviews expired or were suspended. Furthermore, the opportunities for follow-ups with doctors and the possibilities for rehabilitation courses drastically decreased. People with disabilities were also discriminated in accessing treatment and diagnosis due to the poor development of accessible information (Negrini et al., 2020), as well as being heavily penalized by the closure of schools and not always in a position to take advantage of distance learning and teaching. In the period April-June 2020, over 23% of Italian students with disabilities (about 70,000) did not take part in remote lessons, against an average of 8% of other students (ISTAT, 2022). In this regard, one-third of children with disabilities increased their withdrawal behaviors, anxiety-depressive behaviors, attention problems, and aggressions. Finally, as regards the impact on the working situation of people with disabilities in Italy (FISH, 2020), workers with disabilities endured a series of additional inconveniences: 32% of them suspended their jobs, and only 34.3% had access to smart-working. As a result, there was an increase in economic difficulties concerning a reduction in revenues (39%) and greater costs incurred (61%). In the face of these critical issues testified by data presented, it appears that some psychological resources, namely resilience and future orientation (Santilli et al., 2021; Bricout et al., 2022) played a protective role from the disrupting effects of the COVID-19 pandemic. Although the health emergency aggravated and amplified these problems and their consequences, there is a lack of research and scientific studies investigating the incidence of psychological discomfort and overall suffering in people with disabilities, in their families, and the social networks associated with them following the pandemic. On the other hand, it is of primary importance to understand and to research the psychological resources that could contribute to enhancing the quality of life and wellbeing of all the actors involved in the people with disabilities’ community.

### Disability

In the present study disability is considered a long-term impairment resulting from processes of interaction potentially emerging between any individual and the environment, both in a physical and social way, in line with the United Nations International Convention of the Rights of Persons with Disabilities (CRPD). Given the methodological implications of this ontological and epistemological positioning (Guba and Lincoln, 1994), it will herein briefly be described. Since the 2000s, there has been an evident change in how common sense and scientific approaches are built around the theme of disabilities. One of the fundamental events that contributed to this change of direction can be traced to the adoption by the General Assembly of the United Nations of the International Convention on the Rights of Persons with Disabilities (CRPD), ratified by Italy with Law 18 of 3 March 2009. The overall purpose of the CRPD is to “promote, protect and ensure the full and equal enjoyment of all human rights and fundamental freedoms by persons with disabilities, and to promote respect for their inherent dignity” (Article 1). In line with the International Classification of Functioning (ICF), disability is indeed no longer configured as a static, intrinsic, and defining characteristic of the person, but as an evolving concept “resulting of a process of interaction between one or more conditions of the person and the various “attitudinal and environmental” barriers.” Indeed, in Art. 1 paragraph 2 the CRPD specifies that “Persons with disabilities include those who have long-term physical, mental, intellectual or sensory impairments which in interaction with various barriers may hinder their full and effective participation in society on an equal basis with others.” Supporting a perspective in which disability can potentially emerge in anyone’s life through complex interactions between personal characteristics and contextual barriers, the CRPD and the ICF represent a clear end to the logic that considers people with disabilities as a social category. The perspective deriving from the medical-health model in which the person with a disability is to be considered a “sick person” comparable to the condition of a “patient,” which inevitably leads to the broader category of “person in need,” is therefore hopefully outdated. This finding appears in line with the CRPD, which in its preamble and referring to the international context states that persons with disabilities continue to face barriers in their participation as equal members of society (World Health Organization, 2001; United Nation Human Rights Council, 2006). This disparity emerges as a condition of material disadvantage to the detriment of the formal equality pursued by national and international legislation. The elective notion becomes instead the consideration of people with disability as “subjects of right.” It will be necessary to respond to their legitimate expectations as to those of any citizen in global terms of “health, education, work, mobility, participation in the social and political community life, overcoming discriminations and restrictions,” so facilitating “a vision and an organic response to the needs of all people” (Soresi et al., 2016).

### Life Design approach

The Life Design paradigm (Savickas et al., 2009) was developed as a response to a series of rapid social and cultural changes that strongly impacted people’s lives in the twenty-first century. Given the contemporary challenges characterized by a decrease in the wellbeing of individuals, groups, and communities, the Life Design approach abandoned the purely linear and individual analysis perspectives in favor of more “circular” and contextual conceptions. It is a paradigm that emphasizes the continuous evolution of the individual, society, and the modern economy. Life Design epistemology is based on social constructionism, according to which the identity and knowledge of an individual are the product of social interactions (Savickas et al., 2009). Life Design consequently recognizes that the knowledge and identity possessed and developed by a person derive from the social and cognitive processes that take place during the interactions between people and groups, as well as in the negotiations between them (Gasper, 1999). In this perspective, the meaning that a person attributes to reality is therefore co-constructed in a social, historical, and cultural context through the discourses that take place (Young and Collin, 2004). Consequently, unlike in past decades, a person’s development no longer follows linear and predictable trajectories. Today’s global society makes it necessary to know how to manage uncertainty and frequent transitions. To achieve a good life project, individuals need to harmoniously integrate the different contexts of life, work, personal values, expectations, desires, and the multiple roles covered (Savickas, 2012). Life Design addresses people of all ages suggesting ways to give value to such needs, desires, and skills, to make choices, and to make increasingly effective decisions. It provides operational and concrete tools to design educational and professional life by harmoniously integrating the different relative contexts: work, personal values, expectations, desires, and the multiple roles covered. By increasing reflective skills, Life Design favors the development and increase of resources helpful in dealing with the difficulties encountered in life. Furthermore, the paradigm refers to Design Thinking–analytical skills and creative aptitudes–and feeds on good narratives to help people constantly adapt to new contexts, allowing the individual to remain stable while flexibly responding to the demands of the environment and supporting the development of the ability to anticipate changes with the solicitation of proactive behaviors (Cruz Rosas and Oseda Gago, 2022). From the Life Design point-of-view, particular emphasis is given to essential dimensions such as career adaptability, time perspective and future orientation, hope, and resilience. These skills can constitute the guide and the means for dealing with the COVID-19 emergency. In planning and managing one’s life project, Life Design favors flexibility, adaptability, and learning throughout life. It aims to help people outline their history through adaptive responses in order to fulfill their evolutionary tasks and transitions by finding satisfactory solutions for wellbeing and life satisfaction. Within the specific emergency context triggered by the spread of COVID-19, the objectives identified in the theoretical framework aim to enhance the ability to anticipate changes and one’s future in stochastic contexts, finding ways to meet one’s expectations of life, and support individuals in the processes of re-construction and co-construction of their personal and professional trajectories (Savickas et al., 2009). To conclude, the final aim in following this approach was to assess Life Satisfaction. In the literature, life satisfaction is described as a subjective component of quality of life and this concept has assumed an important role in studies on people, including those with disability (Schalock and Felce, 2004; Santilli et al., 2014; Ginevra et al., 2018). Life satisfaction was used in this study for several reasons. First, life satisfaction is largely validated and accepted as a measure of wellbeing, as supported by different studies (Levacher et al., 2023). Secondly, in line with the literature and the Life Design paradigm, life satisfaction helps people deal positively and with awareness in the face of stressful life events, operating as a buffer against some effects of complex life events. In fact, life satisfaction refers to the ability to provide for people’s needs and the opportunity to pursue improvements in the setting of major life activities (Suldo and Huebner, 2004; Pavot and Diener, 2008; Wehmeyer, 2013; Santilli et al., 2014). In various studies, life satisfaction is used as an outcome, given its role in dealing with possible difficult situations and events (Hirschi, 2009; Green, 2011; Maggiori et al., 2013). Lastly, the relevant literature shows how cognitive processing of stressful events, such as COVID-19 pandemic, has a significant impact on life satisfaction and overall wellbeing (Wang C. et al., 2020; Diao et al., 2023). In fact, the implications of the pandemic have had an important effect on the population’s psychological health and wellbeing, regarding for example loneliness, depression, anxiety, and life satisfaction (Bou-Hamad et al., 2021). Thus, considering the objectives of the study, it appears necessary to try to implement life satisfaction given the positive outcomes it has on people’s overall health.

### The DISCOVID research project and research goal

DISCOVID is a national research project conducted in collaboration with the Italian Government (2021–2022) and funded by the Italian Disability Office of the Council of Ministers. Through psychosocial research and from a Life Design Approach perspective (Savickas et al., 2009), the project sought to investigate the impacts of the COVID-19 pandemic on people with disabilities, their families, and the social and association context in the field of disabilities. The investigation started by establishing the needs and criticalities experienced by people with a disability during the lockdown, as well as the effects of the pandemic and the psychological resources that individuals possessed or needed to develop to manage this complex period. With a Life Design approach, the study aimed to increase the understanding of the relationship between career adaptability, future orientation, and resilience on life satisfaction in the COVID-19 period. Based on Ginevra et al. (2018) study, we expected that the capacity to be positively projected into a possible future and exploring the context with confidence (Career Adaptability) would relate positively to satisfaction in one own life. In addition, given previous findings (Camussi et al., 2023), we also expected resilience, namely “the developable capacity to rebound or bounce back from adversity, conflict, and failure or even positive events, progress, and increased responsibility” (Luthans, 2002, p. 72) and time perspective to be related to personal satisfaction, despite personal characteristics such as gender or age (Goal 1). Our second Goal (Goal 2) was to explore possible differences in the dimensions assessed related to the types of disability considered (physical, intellectual, sensory). As a final goal, we expected to provide helpful and practical suggestions to support the development of a broader range of innovative policies. Indeed, a secondary aim was to identify practices, strategies, and social, territorial, family, and personal resources crucial to dealing with the difficulties deriving from COVID-19. A final and implicit aim was to give a voice to people with disabilities, capturing from their experiences and life stories the critical issues that have affected their quality of life and may compromise it in the future (Goal 3). This is a fundamental goal given the absence of research that centered on vulnerable people and their experience during the COVID-19 pandemic. To achieve these goals, we chose a Sequential Explanatory Design for our study (Ivankova et al., 2006). It consisted of integrating both quantitative and qualitative data in the research process, gaining a better understanding of the research theme explored. The reason for mixing both quantitative and qualitative data in a single study lies in the fact that using both methods allow to better encompass the specific trends and aspects of the impacts of COVID-19 on the community of people with disabilities. In fact, when combined, quantitative and qualitative methods are complementary and lead to more robust analysis, exploiting each method’s strengths and mitigating their weaknesses (Creswell and Creswell, 2005).

## Materials and methods

### Quantitative section

In addition to the request for gender, age and disability type (percentages will be described in the section “Participants”), the quantitative section of the questionnaire comprised the measurement of the following listed dimensions:

#### Career adapt-abilities scale–Italian form

This scale (Soresi et al., 2012) aims to measure Career Adaptability–Professional Readiness and Adaptability—and is composed of 24 items equally divided into 4 subscales that reflect adaptability resources: Concern (e.g., “Planning how to achieve my goals”), Control (e.g., “Taking responsibility for my actions”), Curiosity (e.g., “Observing different ways of doing things”), and Confidence (e.g., “Solving problems”). The four subscales combine into a total score, indicating career adaptability. All items are formulated positively and rated using a five-point Likert scale ranging from 1 (I do not possess this competence at all) to 5 (I totally possess this competence). Cronbach’s alpha for this sample is 0.97.

#### Design my future

The scale (Di Maggio et al., 2016) evaluates the presence of positive personal dynamics and optimistic mental dispositions regarding one’s future. These mental dispositions reflect different levels of openness to innovation, exploration of one’s personal and work environment, and ability to create strategies to achieve concrete goals and plans (Di Maggio et al., 2016). It is composed of two subscales: Future Orientation, which refers to the individual propensity to imagine one’s future and to reflect in a hopeful way on one’s life plans (11 items, e.g., “Thinking about the future excites me”), and Resilience, the ability to positively manage adverse events and failures, configuring them as opportunities for growth (8 items, e.g., “Even under pressure, I can concentrate and think deeply and carefully”). The tool comprises 19 items on a 5-point Likert scale (from 1 to 5). Cronbach’s alpha for Future Orientation in this sample is 0.95, while for Resilience is 0.91.

#### Satisfaction with life scale

This scale (Diener et al., 1985) evaluates overall general life satisfaction, a subjective component of quality of life. It does not refer to specific domains such as health or finances, but instead allows the person to weigh these dimensions independently and subjectively. The instrument consists of five items on a five-point Likert scale (from 1 = strongly disagree to 5 = strongly agree). Examples of items are: “I am satisfied with my life” and “If I could live my life again, I would change almost nothing.” Cronbach’s alpha for this sample is 0.89.

### Qualitative section

To achieve the goal of giving a voice to people with disabilities, a space in the questionnaire was dedicated to collecting comments from the participants. To promote voluntary contributions only, a parenthesis below the question reaffirmed that the answer was optional. The question posed, which required an open answer, was stated with the following formulation: “Any Comments (this question is optional).” This kind of formulation, of a neutral nature, was used to avoid possible phenomena of distortion of the spontaneous content brought by the respondents.

### Participants

As a premise, the sampling process for this research, comprising people with disability, was conducted and made possible thanks to the cooperation of the national and local networks of associations dealing with people with disabilities, contacted by mandate of the “Osservatorio Nazionale sulla condizione delle persone con disabilità della Presidenza del Consiglio dei Ministri” (Italian Government National Observatory on the condition of People with Disabilities). The questionnaire sample was composed of 403 people with disabilities, of which 231 were women (56.9%), 166 men (40.9%), 5 people belonging to “other genders” (1.2%), and 1 person who decided not to declare their gender. The age of participants covered different age groups: 148 individuals under 30 (36.5%), 71 individuals between 31 and 40 (17.5%), 62 individuals between 41 and 50 (15.3%), 65 individuals between 51 and 60 (16%), and 50 who were over 61 years old (12.3%). The geographical location of the sample was variegated; 298 people with disabilities are from Northern Italy, 50 are from Central Italy, and 49 are from Southern Italy or the Italian islands. Some of the participants decided not to declare where they lived. A total of 212 people were people with a physical/motor disability (52.3%), 130 were people with a cognitive/intellectual disability (32%), and 61 were people with a sensory disability (15%). Considering the Educational Level, 6 people with disabilities have a Primary School Diploma (1.5%), 71 people have a Middle School Diploma (19.5%), 201 individuals have a High School (49.5%), 36 people have a bachelor’s degree (8.9%), 46 people have a master’s degree (11.3%), and 15 people with disability have a Post Graduated level (3.7%). Regarding the Job Status, 61 (15%) people with disability were students, 130 (32%) were workers, 38 (16.7%) were retired, and 76 (18.7%) were unemployed people. As regards marital status, 260 participants were single (64%), 89 of them (21.9%) were married or in a stable relationship, 23 (5.7%) were divorced, and 11 people with disability (2.7%) were widower. Some of the participants decided not to declare this information. Gender, age, disability type, geographical related information, educational level, job situation, and marital status were obtained by direct question inside a specific section of the questionnaire.

### Procedure

The data was collected with an online questionnaire. The questionnaire was distributed across Italy through connections with national associations and people working in the disability field. All participants were obliged to answer all the items, except for the final open question. The entire questionnaire lasted approximately 30 min. Each participant was informed of the absolute anonymity of the research, obtained through the omission, in the reports, of any element that could lead to identification. Informed consent and consent to data processing for research purposes were obtained from all participants. The study was conducted in line with the ethical guidelines of the Italian Society for Vocational Guidance (SIO), the Declaration of Helsinki, and the Oviedo Convention. Moreover, the research was approved by the Ethical Committee of Milano-Bicocca University.

### Data analysis

One of the purposes of the study was to investigate the possible influence of personal resources (Career Adaptability, Resilience, Future Orientation) on satisfaction with one’s life, controlling the impact of the socio-demographic variables (such as age, gender, and typology of disability, educational level, marital status, and job situation). Indeed, according to the literature, socio-demographic characteristics and identity belonging could influence life satisfaction perception (Sarriera et al., 2014; Dos Santos et al., 2019). For this reason, hierarchical regression was chosen: a type of regression in which predictors are introduced in steps or blocks. It is thus possible to consider each block as one model. The first block introduced the control variables, which it is necessary to hold constant (such as age, gender, typology of disability, educational level, marital status, and job situation). The aim was to handle the changeability of the control variables by extracting them before analyzing the relationship between the personal resources–as predictors–and the satisfaction with life–as the outcome (Fein et al., 2022). Indeed, this analysis model could permit to control the effect of category belonging on perceived levels of life satisfaction (Fernández-Ballesteros et al., 2001; Benke et al., 2020; Karataş et al., 2021; Karabağ Aydın and Fidan, 2022). At the same time, in line with studies of Hirschi (2009), Scioli et al. (2011), Maggiori et al. (2013), and Santilli et al. (2014) that observed direct relationships between personal resources, such as Career Adaptability, and life satisfaction, the hierarchical multiple regression could measure the role of these resources in predicting life satisfaction, about the issue of disability and the pandemic context.

Quantitative data analyses were performed with IBM SPSS Statistics (Version 28). Several preliminary analyses were conducted before analyzing the relationship between personal satisfaction and skills. First, missing responses, skewness, and kurtosis were assessed. Second, the normality of distributions of personal satisfaction, future orientation, career adaptability, and resilience was tested using Kolmogorov-Smirnov statistic. Third, all means, standard deviations, and Pearson correlations were performed on the study variables. Finally, as the fourth step, one hierarchical multiple regression analysis (Stone-Romero and Anderson, 1994) was carried out to control the effect of the socio-demographic variables (age, gender, and typology of disability) and to determine the effect of personal resources (Career Adaptability, Resilience, Future Orientation) on satisfaction with one’s life. In order to test the predictions, the hierarchical multiple regression was conducted with three blocks of variables. Demographic variables (Gender, Age, and Disability Type) were entered in step 1 of the regression model. The second block included educational level, job situation, and marital status as the predictors, with Satisfaction with Life as the dependent variable. In block three, levels of personal resources (Career Adaptability, Resilience, Future Orientation) were also included as the predictor variable, with Satisfaction with Life as the dependent variable (Goal 1, Goal 2). ANOVA (Goal 2).

Finally, regarding the different effects of gender, age, disability type, educational level, job situation, and marital status on Personal Satisfaction and Smart skills, the different levels of Personal Satisfaction, Future Orientation, Career Adaptability, and Resilience, were analyzed with a one-way linear ANOVA (Goal 2).

The content of the final answer (qualitative data) provided was subjected to the thematic analysis method to understand and systematize the contents that emerged (Goal 3). This methodology is based on the search for themes emerging from the data. It considers language as constitutive of meanings and the construction of meanings as “the outcome of social processes.” The following analysis was inspired by the methodological features discussed by Braun and Clarke (2006) in “Using thematic analysis in psychology.” The procedure involves several steps. The first step comprises data familiarization through repeated reading of the material, making it possible to become acquainted with the variety in the content of the answers. The second step was to establish the main themes explicitly expressed by the respondents, to identify the most frequent recurring concepts and, consequently, to create “verbal categories” to identify aspects of similarity between the answers. Finally, the labels assigned were categorized into thematic families. Superordinate conceptual groupings were established to combine several labels with analogous reference concepts.

## Results

Table 1 reports all means standard deviations for the whole sample and the sample divided by the demographic characteristics. Regarding exploring possible differences in the levels of smart skills between different types of disability (Goal 2), the ANOVA analysis revealed that the different types of disabilities significantly impact Personal Satisfaction, Career Adaptability, and Resilience. Indeed, there was a significant effect of type of disabilities on Personal Satisfaction *F*_(2, 400)_ = 12,033, *p* < 0.001 and on Career Adaptability *F*_(2, 400)_ = 13,381, *p* < 0.001. Specifically, it was found that people with sensory disabilities had significantly higher levels of Career Adaptability (*M* = 3.722; SD = 0.776) and Resilience (*M* = 3.379; SD = 0.884). People with intellectual disabilities showed significantly lower levels of smart skills (Career Adaptability *M* = 3.155; SD = 0.962; Future Orientation *M* = 3.055; SD = 1.039; Resilience *M* = 3.069; SD = 0.952) but a higher level of Personal Satisfaction (*M* = 3.107; SD = 0.917). People with physical disabilities showed a medium level of smart skills (Career Adaptability *M* = 3.625; SD = 0.895; Resilience *M* = 3.271; SD = 0.997; and Future Orientation *M* = 3.007; SD = 1.062) and a lower level of Personal Satisfaction (*M* = 2.603; SD = 0.948).

**TABLE 1:** Means and standard deviations; *T*-test and ANOVA analysis of the variables with characteristics of participants.

		1. Personal satisfaction	2. Future orientation	3. Career adaptability	4. Resilience
Whole sample	*M* ± *SD*	2.813 ± 0.964	3.054 ± 1.057	3.491 ± 0.934	3.224 ± 0.977
**Type of disabilities**					
Physical disability	*M* ± *SD*	2.603 ± 0.948	3.007 ± 1.062	3.625 ± 0.895	3.271 ± 0.997
Cognitive disability	*M* ± *SD*	3.107 ± 0.917	3.055 ± 1.039	3.155 ± 0.962	3.069 ± 0.952
Sensory disability	*M* ± *SD*	2.901 ± 0.941	3.192 ± 1.031	3.722 ± 0.776	3.379 ± 0.884
F		12.033**	0.736	13.381**	0.934
**Gender**					
Female	*M* ± *SD*	2.680 ± 0.965	3.003 ± 1.070	3.514 ± 0.923	3.200 ± 0.973
Male	*M* ± *SD*	3.013 ± 0.931	3.112 ± 10.028	3.442 ± 0.940	3.253 ± 0.980
t		−3.346**	−1.019	0.725	−0.537
**Age**					
<30	*M* ± *SD*	2.968 ± 1.009	3.303 ± 0.822	3.506 ± 1.001	3.237 ± 0.907
31–40	*M* ± *SD*	2.727 ± 0.974	2.992 ± 0.912	3.303 ± 0.926	3.099 ± 0.876
41–50	*M* ± *SD*	2.665 ± 0.1.010	2.881 ± 0.935	3.737 ± 1.120	3.373 ± 1.043
51–60	*M* ± *SD*	2.695 ± 0.868	2.831 ± 1.034	3.370 ± 1.030	3.171 ± 1.077
>60	*M* ± *SD*	2.756 ± 0.886	2.855 ± 1.061	3.488 ± 1.170	3.223 ± 1.063
F		1.747	3.862*	2.100	0.584
**Educational level**					
Primary school	*M* ± *SD*	2.300 ± 1.108	1.924 ± 1.120	2.465 ± 1.062	2.208 ± 1.020
Middle school	*M* ± *SD*	3.139 ± 0.908	2.983 ± 1.064	3.118 ± 1.039	2.962 ± 1.074
High school	*M* ± *SD*	2.838 ± 0.948	3.080 ± 1.013	3.490 ± 0.847	3.254 ± 0.881
Bachelor’s degree	*M* ± *SD*	2.367 ± 0.868	3.048 ± 0.920	3.789 ± 0.798	3.319 ± 0.900
Master’s degree	*M* ± *SD*	2.657 ± 0.922	3.063 ± 1.149	3.730 ± 0.906	3.299 ± 1.023
Post graduated	*M* ± *SD*	2.650 ± 1.482	2.591 ± 1.852	3.344 ± 1.674	3.000 ± 1.655
F		3.096*	1.452	5.217**	3.220*
**Job situation**					
Students	*M* ± *SD*	2.662 ± 0.914	3.210 ± 1.038	3.563 ± 0.909	3.148 ± 1.022
Workers	*M* ± *SD*	2.908 ± 0.893	3.00 ± 1.025	3.635 ± 0.853	3.385 ± 0.942
Retired	*M* ± *SD*	2.753 ± 1.000	2.941 ± 1.144	3.362 ± 1.040	3.105 ± 1.041
Unemployed	*M* ± *SD*	2.921 ± 1.052	3.181 ± 1.036	3.467 ± 0.889	3.183 ± 0.897
F		1.148	0.729	1.624	1.264
**Marital status**					
Single	*M* ± *SD*	2.850 ± 0.983	3.057 ± 1.024	3.370 ± 0.925	3.116 ± 0.946
Married/In a stable relationship	*M* ± *SD*	2.883 ± 0.856	3.028 ± 1.026	3.748 ± 0.812	3.502 ± 0.916
Divorced	*M* ± *SD*	2.357 ± 1.136	3.114 ± 1.296	3.940 ± 0.834	3.533 ± 1.067
Widower	*M* ± *SD*	2.436 ± 0.933	2.479 ± 1.361	2.780 ± 1.440	2.977 ± 1.438
F		2.006	1.132	6.459**	3.530

**p < 0.001; *p < 0.05.

Data show that men have higher life satisfaction *F*_(395, 362)_ = 1,495, *p* < 0.001 (men: *M* = 3.013; SD = 0.931; women: *M* = 2.680; SD = 0.965), as do people with middle school diploma [*F*_(395, 362)_ = 1,495, *p* < 0.001]. Specifically, it was found that people with a middle school diploma show higher satisfaction about their life (*M* = 3.139; SD = 0.908), followed by the people with a master’s degree (*M* = 2.657; SD = 0.922) or a post-graduation path (*M* = 2.650; SD = 1.482). People with high school diploma, bachelor’s degree, and primary school diploma showed lower satisfaction about their life, with *M* = 2.838; SD = 0.948; *M* = 2.367; SD = 0.868, *M* = 2.300; SD = 1.108, respectively. The educational level is also connected with Career Adaptability *F*_(7, 395)_ = 5,217, *p* < 0.001 and Resilience *F*_(7, 395)_ = 3.220, *p* = 0.002. People with a bachelor’s or master’s Degree (3.789 ± 0.7983.730 ± 0.906), a high school diploma (*M* = 3.490; SD = 0.847) or a post-graduated certification (*M* = 3.344; SD = 1.674) showed a higher level of the capacity to control the future, have curiosity, be confident, and act with concern (Career Adaptability). People with a middle school (*M* = 3.118; SD = 1.039) or a primary school diploma (*M* = 2.465; SD = 1.062) showed less Career Adaptability. In addition, people with a primary school or middle school diploma showed less resilience (*M* = 2.208; SD = 1.020; *M* = 2.962; SD = 1.074, respectively) than people with a high school diploma (*M* = 3.254; SD = 881), a degree (*M* = 3.319; SD = 900; *M* = 3.299; SD = 1.023) or a post-graduation path (*M* = 3.000; SD = 1.655). There was a significant effect of age on Future Orientation *F*_(4, 391)_ = 3,862, *p* = 0.004. Specifically, it was found that younger people had significantly higher levels of Future Orientation, connecting past, present, and future, too (e.g., people under 30 years old *M* = 2.968; SD = 1.009 and people between 51- and 60-years old *M* = 2.695; SD = 0.868). The last significant ANOVA was about the effect of the marital status on Career Adaptability *F*_(4, 398)_ = 6,459, *p* < 0.001. People with stable relationships or divorced showed significantly higher levels of Career Adaptability (*M* = 3.748; SD = 0.812; *M* = 3.940; SD = 0.834, respectively) than single (*M* = 3.370; SD = 0.925) and widower (*M* = 2.780; SD = 1.440).

Table 2 reports the correlation between personal Satisfaction and Career Adaptability, Future Orientation, and Resilience.

**TABLE 2:** Correlations among personal life-satisfaction and smart skills.

	1	2	3	4
1. Personal satisfaction	1	0.424**	0.198**	0.343**
2. Future orientation		1	0.636**	0.693**
3. Career adaptability			1	0.740**
4. Resilience				1

**p < 0.01.

Considering our goal (Goal 1) to analyze the relationship between smart skills and personal life satisfaction during the pandemic while taking account of the potential influence of personal characteristics (Table 3), the hierarchical multiple regression showed that Model 1 with demographic variables was significant [*F*_(3,392)_ = 5.212 Δ*R*^2^ = 0.042, *p* = <0.001]. Specifically, the variable “Gender,” and “Type of disability” positively influence personal life satisfaction (β = 1.00, *t* = 1.995, *p* < 0.001, and β = −1.40, *t* = 2.723, *p* < 0.005, respectively). Meanwhile, Age is not significant as reported in Table 3. The second model [*F*_(3,389)_ = 3.870, Δ*R*^2^ = 0.014 *p* = 0.128], which included Educational Level (β = −0.110, *t* = −2.189, *p* = 0.029), Job Status (β = 0.011, *t* = −0.228 *p* = 0.820), and Marital Status (β = −0.044, *t* = −0.849, *p* = 0.396) did not show significant improvement from the first model. Moreover, model 3 with personal skills had a significant improvement (Δ*R*^2^ = 0.87 <0.001). Career Adaptability positively (β = 0.175, *t* = 2.533, *p* < 0.05) predicted personal satisfaction during the pandemic period. In addition, the capacity to withstand difficulties, cope, and face complexity during COVID-19 (Resilience) was found to be significant (β = 0.238, *t* = 3.305, *p* = 0.001). The same was true for Future Orientation, the capacity to think about the past, present, and future, anticipating future consequences (β = 0.374, *t* = 5.790, *p* < 0.001).

**TABLE 3:** Hierarchical multiple regression analyses predicting personal life-satisfaction.

Predictor	Δ*R*^2^	β
Step 1	0.042**	
Gender		0.100*
Age		-0.064
Type of disability		0.140*
Step 2	0.014	
Educational level		-0.110*
Job situation		-0.011
Marital status		-0.044
Step 3	0.198**	
Career adaptability		0.175*
Future orientation		0.374**
Resilience		0.238**

**p < 0.001; *p < 0.05.

Overall, when only gender, age, and type of disabilities were included in the model, the variables explained 4.2% of the variance, with the final model, including personal skills accounted for 25.5% of the variance.

Regarding the qualitative analysis carried out on the final optional question (Table 4), various categories emerged and will herein be described. In some cases, these categories were strictly semantically related and reflected similar positionings. Sometimes they were quite opposed, reflecting not only different ideas and positionings, but also participants’ different focalizations while answering, e.g., with a more external or internal point of view. This resulted in both participants directly addressing their ideas on COVID-19’s consequences on them, on closed ones, on the world and society in general, or not addressing COVID-19 at all. Given the variety and multitude of comments, these were summarized into 6 main thematic families and 11 thematic subcategories (Goal 3). Table 3 also reports a quantification of the comments by the category in which they were included after the analysis procedure.

**TABLE 4:** Families and categories from the optional comments.

Family name	Subcategory name	% of the category	% of the families
Initiative approval	Thanks	11.36%	31.81%
	Positive evaluation	18.18%	
	Request for a report	2.27%	
Questionnaire criticism		20.45%	20.45%
Description of personal history	Description of type of disability	20.45%	22.72%
	Territorial specifics	2.27%	
COVID-19 and repercussions		9.09%	9.09%
Possible actions	Concrete actions by institutions	9.09%	26.99%
	Enforcement of rights	6.82%	
	More work	4.54%	
	Future and time perspective	4.54%	
Non-codifiable	No	4.54%	9.08%
	Other	4.54%	

The first thematic family, called “Initiative approval,” comprises all the content in which people express their support for the research. Within it, 3 subcategories have been grouped together, namely “Thanks” (e.g., “*Thank you for this questionnaire. I hope it bears fruit. Best regards*.”–comment No 9, woman, 46–50, person with motor disability), “Positive evaluation” (e.g., “*An excellent job I hope it could be useful to improve the social situation*”–comment No 3, man, 60–65, person with motor and sensory disability), and “Request for a report” (e.g., “*At the end of the questionnaires and related analysis, if you can, can you send me the related results?*”–comment No 41, man, 71–75, person with motor disability).

The second thematic family was called “Criticism of the Questionnaire,” and it collects all the difficulties and other generic judgments regarding the questionnaire, its items, the research, or references to possible theoretical negligence deduced from the questionnaire. One example is “*The questions are numerous, and the questionnaire was long*” (comment No 22, man, 41–45, person with sensory disability).

The third thematic family was named “Description of personal history,” and it considers all the comments in which the participants refer to some aspects of their personal history. It is possible to trace two subcategories: “Description of type of disability” (e.g., “*Disability: Asperger’s syndrome*” —comment No 37, man, 26–30, person with autism spectrum disabilities), and “Territorial specifics” (e.g., “*I tried to answer the more general and less personal questions based on my knowledge of Italy. I was born and raised in Italy*”–comment No 30, woman, 31–35, person with motor disability).

The fourth thematic family, called “COVID-19 and repercussions,” considers all the comments that refer to the COVID-19 pandemic and its social, health, psychological, physical, and emotional repercussions on the lives of people with disabilities. Some examples of statements in this category are: “*The State has not helped disabled people*”–comment No 20, man, 46–50, person with motor disability; “*Covid, for now, has left only tiredness and apathy*”–comment No 27, woman, 61–65, person with motor disability; and “*After the pandemic, I was unable to walk and see, now that my mom has been gone, I’m stuck, and I can’t take care of myself, have fun and get around. the caregiver has fulfilled her duty with love, but I lost autonomy, amen*” (comment No 36, woman, 71–75, person with motor and sensory disability).

The fifth thematic family has been entitled “Possible actions”. It refers to possible actions and strategies that people and the community can implement to ensure a higher level of wellbeing for people with disabilities. It includes four subcategories, namely “Concrete actions by the institutions” (e.g., “*Do something concrete for people with disabilities. My negative answers are due to the lack of personal assistance in Italy. At the moment, I cannot foresee a future in which my only guaranteed right is to be institutionalized. People with disabilities must live in society because we don’t bother anyone*”–comment N°20, woman, 20–25, person with motor disability; “*Make us feel alive*”–comment No 33, man, 51–55, person with motor disability); “Enforcement of rights” (e.g., *“Fibromyalgia must be included in the LEA and be officially recognized as a primary disabling pathology. We can no longer bear having to have other related pathologies to be recognized”*–comment No 23, woman, 46–50, person with motor disability; “*I would like accessibility rights to be taken seriously and feel as human rights as they are. I would like ableism to be fought like racism, sexism, homo-lesbo-transphobia. I want to be free to take a taxi at 9 pm and return at 3 am, which is impossible because ramp taxis don’t travel at night ‘due to low demand’* […]. *We are people with a disability, not disabled people*”–comment N°2, gender not specified, 20–25, person with motor disability); “More work” (e.g., “*It would be nice if disabled people were helped more in job placement, I’m still looking for a job, that’s not how you have a life*”–comment No 10, woman, 31–35, person with motor disability); “Future and time perspective” (e.g., “*I hope that we can go toward a better world with lots of Love, Solidarity, and Respect. If united, the world could produce well-being for all!*”–comment No 6, woman, person with motor disability).

The last family is composed of non-codifiable comments, such as *“NO”* (Comment No 16, man, 51–55, person with cognitive disability) or other kinds of comments, such as *“///”* (Comment No. 44, women, 51–55, person with motor disability).

The most frequent families were “Initiative approval” (31.81%), “Possible actions” (26.98%), and “Description of personal history” (22.72%). On the other hand, the “Criticism of the questionnaire” makes up 20.45% of the answers, while “COVID-19 and repercussions” represents 9.09%. Finally, the “Non-codifiable” category accounts for 9.08% of the total labels.

## Discussion

The COVID-19 pandemic had profound and transversal impacts on people all over the world. In addition, national and international data has highlighted that people already facing difficulties, such as those with disabilities, were affected more than the general population by the pandemic (United Nations High Commissioner for Human Rights, 2020; World Health Organization, 2020; ISTAT, 2022). People with disabilities are among those who have borne the heaviest brunt of this situation, suffering repercussions on the sphere of psychosocial wellbeing (Holm et al., 2022), at the economic level (Banks et al., 2023), at the employment level (Jetha et al., 2023), and on their own self-determination and social inclusion (Courtenay and Perera, 2020). Anticipating these scenarios, Turk and McDermott (2020), as well as Reed et al. (2020) have emphasized the need to generate knowledge and data concerning the interaction between COVID-19 and the disability world, so that a disability-inclusive response to health emergencies can be provided. That being said, there is no extensive research in Italy on the psychological conditions of people with disability during and right after the pandemic period. As a premise, it is important to state that, even in the present research, the results that are presented could stem from the interaction of global processes specifically related to the pandemic context, could be specific to the global setback described as a premise for the Life Design approach, and could be true of individuals with disability more generally. In particular, in this paper, attention was paid to life satisfaction of people with disabilities. Following a Life Design perspective, satisfaction with life is considered an important indicator of people’s quality of life (Schalock and Felce, 2004; Santilli et al., 2014). For this reason, as previously explained, this outcome was chosen and the potential protective role of psychological skills and factors, namely career adaptability, resilience and future orientation was studied. Through a quantitative self-report survey administered to people with disabilities in Italy, the present work analyzed the impacts of the COVID-19 pandemic on their life and wellbeing. The aim of the research, from a Life Design perspective (Savickas, 2012) and in line with other Life Design perspective research on the topic (Ginevra et al., 2018) was to better understand how Italian people with disabilities perceived the pandemic emergency, the difficulties they encountered, and how they coped with their needs in the face of their internal and external resources [Goal 1, Goal 2. ANOVA (Goal 2)]. As can be seen from the results obtained through the survey conducted, the resources assessed (career adaptability, resilience and future orientation) were all linked to the personal perceived life satisfaction of those who answered the questionnaire, while assessing and controlling the effect of variables such as age, gender, educational level, job situation, marital status, and type of disability. In fact, although participants’ gender, age, education, job situation, marital status and type of disability concurred in varying their perceived level of psychological resources aimed at managing the complexity of contemporary world and of the pandemic period, people’s ability to adapt, to resist and to design their future were found to be significantly related to their life satisfaction (Goal 1). Although an inquire on smart skills’ levels on people without disabilities was not part of this study, these results are in line with Holm et al. (2022) findings, whose work published in 2022 showed that people with disabilities reported more often than people without disabilities that the pandemic emergency reduced their hope for the future. The significance of this from a psychosocial point-of-view is very serious when compared, for example, to the increase in requests by family members of people with disabilities for discontinuation of life-saving therapies during the pandemic period as a result of a perceived excessively poor quality of life (Chen and McNamara, 2020). These results show the importance of preserving and promoting wellbeing by promoting satisfaction with life, a subjective perception of living a life that’s worth holding on to and fighting for. In order to do so, the positive relation between life satisfaction and resilience, future orientation and career adaptability in people with disabilities even in times of emergency like the one addressed by this study should be kept in consideration. It is thus suggested the importance of promoting those psychological resources in people with disabilities both by further expanding knowledge and experimenting and perfectioning interventions aimed at increasing their levels, which contribute to preserving life satisfaction and wellbeing. Regarding the differences in psychological resources’ levels in people with different disability types [Goal 2, ANOVA (Goal 2)], even if literature assessing this issue is still very limited (Scheffers et al., 2020), our study that finds lower resilience levels in people with intellectual disability is in line with Scheffers et al. (2020) conclusions that “this finding is striking since people with intellectual disability are at a higher risk of experiencing adversity, but resilience can be a buffer to diminish negative effects” (Vervoort-Schel et al., 2018). Concurrently, in the present study, people with disabilities played an active part and took advantage of the possibility to express their present and future needs, clearly stating how deeply and broadly the pandemic impacted their lives (Goal 3). People who participated in the research highlighted how the advent of the COVID-19 pandemic contributed greatly to exacerbating an already difficult living situation. The scarcity of services available to meet needs, which the emergency has further compromised, has undermined respondents’ perceptions of the possibility of living a fulfilling life. The considerations proposed by Leocani et al. (2020) in a study of the Italian population with disabilities during the COVID-19 pandemic appear to correspond. According to these authors, the postponement of neurological rehabilitation activities due to the impelling health crisis and the deprivation of daily activities such as work and school would lead to scenarios of difficult management. Negrini et al. (2020) were of the same opinion, pointing out that the impact of the COVID-19 pandemic, with the postponement of rehabilitation activities and the general decrease in normal occupations, could, on the one hand, worsen the health of people with disabilities and, on the other, exacerbate care burdens on their families. During the pandemic, the precarious balance regarding disability on which the national welfare system rests was made clear, with the closure and restricted access, sometimes temporary, often indefinitely, of associations and services dedicated to people with disabilities and their families. People have found themselves living in isolation, which has contributed to worsening their physical and psychological wellbeing (Bosisio Fazzi et al., 2022). In line with the findings of research that has sought to describe the possibility of access to services and attendance at specialized treatment and rehabilitation centers by people with disabilities, the picture described by the present survey provides a clear representation of the perceptions of those involved concerning the account of their needs during the pandemic emergency. The measures implemented for people with disabilities would have, on the one hand, reflected little understanding of the actual needs of these people, or, on the other hand, were almost absent. A study by Faccioli et al. (2021) of Italian adolescents with disabilities and their parent’s reports (2021), for example, that they would have liked more remote support from both school and healthcare professionals. Also evident was the lack of economic facilitation provisions supporting people with disabilities, who were already orphaned of their references, and grappling with the difficulties arising from isolation in interaction with their daily needs. Indeed, the closure of centers and associations has occurred in parallel with an increase in the economic outlay that burdens families’ monthly budgets, as highlighted by Courtenay and Perea in their study of the impacts of the pandemic on people with intellectual disabilities and their families (2020). An additional difficulty highlighted by people with disabilities was the absence of waivers that would allow them, in some situations, to meet any needs and necessities that were not codified legislatively. If it could be relatively easy for people without disabilities to plan to go out, the same cannot be said for people with disabilities. In many cases, they require special external support, often from another person, not envisaged by the Law Decrees unless through some municipal or regional measures or, at the national level, toward the end of the emergency. These hardships were further exacerbated, as reported by Dror et al. (2021), by the difficulty in accessing information regarding regulations, particularly for people with disabilities. This overall picture thus shows how, in general, the measures for people with disabilities were considered insufficient by people with disabilities themselves, as they did not consider their real needs. The results of the present work also show how, in the face of a generalized criticality regarding external resources, services centers, and associations that were unreachable, closed, or inaccessible for long periods, some people with disabilities have exploited internal resources. These resources play a key role, especially in times of emergency (Camussi et al., 2023). Possessing a good capacity for future orientation which ensured a future-oriented outlook supported personal and professional planning maintenance. This made it possible to maintain a route that did not stop at the difficulties experienced during the hardest periods of the emergency. A view that went beyond and kept a focus on what one would want to do next rather than on what one could not (anymore) do at the time. For those who could exhibit this future orientation, it meant greater adaptability, greater resilience, and, in general, a better state of wellbeing (Santilli et al., 2021). The strong criticality framed by the results, however, highlights the fact that all of this has been and is still delegated to individuals with disabilities or, at most, to their families. This results in a reality that strongly discriminates against those who, for various reasons, have no family or personal resources to call upon, thus finding themselves in a condition of inevitable and further fragility.

### Implications for practice

Starting from the results and conclusions described above, some worksheets were prepared that constitute intervention proposals. The worksheets herein presented are new proposals in line with “Iniziative per il Rilancio” “Italia 2020–2022” (Initiatives for the recovery “Italy 2020–2022,” Comitato di esperti in materia economico-sociale, 2020), a strategic plan for national recovery after COVID-19 pandemic drafted by a task force of experts in different fields appointed in 2020 by Italian Prime Minister. As stated in this plan and as the results suggest, the context should consider identifying innovative strategies and policy solutions to support people with disabilities, their families, and the associations and communities of reference. As a matter of fact, even considering the absence of a direct measurement of the COVID-19 related stressors, as it will more extensively be explained in the limitations and future perspective paragraph, results of this study both contribute to widen knowledge about psychological resources and life satisfaction in the Italian context of people with disabilities, and show the important protective role played by those resources in order to preserve the subjective perception of living a fulfilling and satisfying life for the participants. For each point identified by combining and interpreting qualitative and quantitative results presented, central nodes have been developed from which strategies, interventions, and resources–social, territorial, and personal–derive that can be implemented to deal with the difficulties and complexities linked to and emanating from COVID-19. First, data collected reveal great suffering from the isolation imposed by the lockdown. For people with disabilities more than for others, this meant deteriorations at the physical level and in autonomy, great loneliness and, often, psychological difficulties. Indeed, despite the resources on the ground–social welfare services, associations, institutions, etc., –should be a fundamental point of reference for people with disabilities, their families and caregivers, they have often been perceived as absent, not sufficient and not adequate to the situation of great emergency. Hence the need to rethink the organization of services for people with disabilities and their families, according to a general model of functional proximity welfare, aimed at to increase the psychological resilience of individuals and communities. It emerged the need to create proximity territorial welfare (Camussi et al., 2021) to improve personal wellbeing and social capital for the community. This could be constituted by public structures close to the people. These structures, with their services, could become points of reference and support for people with disabilities, their families, and the entire community. Translated into action, it means to plan at the national level the implementation of an extensive network of proximity welfare garrisons. Those, in addition to providing a range of services aimed at people with disabilities and their families, should also be physical places for meeting, socializing and sharing. Also, research data indicate that in many cases in which “system and institutional” responses have been considered inadequate, the sense of abandonment was partly alleviated by supportive actions and interventions put in place autonomously by non-institutional private and/or voluntary actors. In the emergency phase, these forms of active citizenship have carried out a valuable activity of proximity information, networking, material and relational support of the citizens in fragile conditions. To systematize and enhance the role of voluntary organizations, which in Italy actively involve about 3 million people including employees and volunteers, as a strategic proximity resource, to better target their contribution in emergencies as in the management of everyday life. Moreover, in line with the Life Design Approach, these services could become crucial in building paths aimed at enhancing the ability to anticipate changes and one’s future in stochastic contexts (e.g., Career Adaptability) and find ways to meet people’s personal and professional life expectations, considering individual differences, such as disabilities, in interaction with life contexts. In addition, it is essential to design and develop expert training courses. The training, which must be managed by senior guidance experts (with specific additional training in this area), could extensively use technology for webinars, podcasts, and platforms to share materials and projects, combined with some essential face-to-face laboratory sessions. In parallel, it becomes even more crucial to improve psychological support services and face-to-face and online interventions to deal with the high growth of anxiety situations, depression, sleep disturbances, and relational problems, and to prevent and reduce depressive syndromes and the connected social and health costs. At last, most of these proposals, as long as many of the structural improvements Italy saw during the pandemic period, dealt with technology. Yet, a great portion of Italian population still struggle with access to technology, both on a hardware level (e.g., lack of devices) and on a software level. Actively tackling the digital divide still existing in Italy could greatly and transversally improve the wellbeing of Italian population.

### Limitations and future research perspective

In order to better comprehend and position this study within the broad frame of the literature concerning COVID-19 and its effects, this paper’s limitations must be considered. First, this study’s aim to inquire and better understand the effects of COVID-19 pandemic on people with disabilities as the protective role played by resilience, career adaptability and future orientation for the life satisfaction of people with disabilities, was not extended to the general population. This means that, having no comparison group and only being able to frame our findings in the light of national and international literature about these themes, statements regarding people with disabilities could also apply to people without disabilities and the population in general, and this should be explored in future research. Indeed, as highlighted in the discussion paragraph, the lack of a direct comparison group means that this study is not able to state whether the observed effects are specific to the pandemic context, specific to some kind of global setback, or true of individuals with disabilities more generally. In future studies, besides to the presence of a comparison group, also the effect of specific variables (as for example related to the labor market situation, job search, sense of anomy or uncertainty, etc.) is to be explored, to better assess and measure the impact of the pandemic on specific domains as long as to find more targeted ways to promote people with disabilities’ wellbeing. In order to do so with incremented validity and precision, the interaction of more specific and transversal factors should also be studied in the frame of other wellbeing theories and factors, with the aim of constructing valid explicative and intervention models. In addition to this, despite the inclusion in this study of a differentiation between types of disabilities, a direct measurement, and consequently a comparison, between different severity levels of disability was not included and should be explored in the future. Moreover, this study assessed the role of resilience, future orientation and career adaptability in protecting satisfaction with one’s life of people with disabilities during the COVID-19 pandemic. Nevertheless, one of this study’s limitations lays in the fact that it does not include any direct ways of measuring any kind of psychological distress levels, nor other measures regarding the effects of the pandemic period. The explorative study herein proposed should be implemented with further research in the future which should address and study specific COVID-19 related stressors, as perceived loneliness, sense of isolation and of abandonment, etc., assessing the magnitude of these COVID-19 related stressors and the interaction between these, protective factors and life satisfaction. As a matter of fact, even the outcome variable chosen, namely satisfaction with life, has some limitations. First, it was a single variable regarding wellbeing in the light of Life Design approach, but it could have been implemented with other theoretical frameworks and/or measurements, such as hedonic and eudemonic wellbeing. Second, other variables regarding potential protective factors and psychological resources, both in a Life Design and in other perspectives, could have been explored, such as Hope, Optimism, Courage, Communication Skills, together with other measures both for psychological wellbeing and distress. As a result of these first and second points, it was not possible to extract an explicative model from the data collected and the analysis performed. Third, given the methods of measurement chosen, personal and situational biases in the response, such as social desirability, could have occurred and could not be fully controlled. Future research could consider multiple methods, such as enhanced qualitative enquiries and/or other direct or indirect quantitative methods, to reduce the influence of the bias due to self-reporting and to consider other personal or situational factors, e.g., the social and family influences on these variables. As a matter of fact, future research may be prompted to adopt longitudinal approaches, to examine if, for example, career adaptability and courage may help to facilitate the management of new emerging challenges and potentially constant uncertainty feelings and positively affect the levels of life satisfaction across months, years or age ranges. Regarding the implications for practice presented here, suggestions have been formulated starting from the results obtained both in the qualitative and quantitative sections of this study, but some limitations should be highlighted. First, the intervention proposals cannot directly stem from what participants stated, as there was not a direct question asking them what should have been done during the pandemic or should be done in the future. Second, the intervention proposals were framed within a framework of previously yet newly stated proposals to broadly enhance general population’s wellbeing and recovery after COVID-19 pandemic, suggested by a scientific experts’ task force nominated by Italian Government (Comitato di esperti in materia economico-sociale, 2020). For these reasons the formulated proposals cannot be compared to similar interventions performed in the past. Future research might focus more directly on understanding the needs of people with disabilities and proposing interventions to enhance their wellbeing.

## Data availability statement

The raw data supporting the conclusion of this article will not be made publicly available due to concerns regarding participant anonymity. Requests to access the datasets should be directed to the corresponding author.

## Ethics statement

The studies involving human participants were reviewed and approved by the Ethical Committee University of Milano-Bicocca. The participants provided their written informed consent to participate in this study.

## Author contributions

EC: supervision (lead), and conceptualization (equal). DM: conceptualization (equal), writing – original draft (equal), and writing – review, and editing (supporting). MLS: writing – original draft (equal), and writing – review and editing (supporting). RR: writing – original draft (equal) and writing – review and editing (equal). FB: writing – original draft (supporting) and writing – review and editing (supporting). CS: writing – original draft (supporting) and writing – review, and editing (supporting). LM: writing – original draft (supporting). CA: conceptualization (lead), writing – original draft (lead), methodology and formal analysis (lead), and writing – review, and editing (lead). All authors contributed to the article and approved the submitted version.
